# Genetic Variants in Nuclear-Encoded Mitochondrial Genes Influence AIDS Progression

**DOI:** 10.1371/journal.pone.0012862

**Published:** 2010-09-21

**Authors:** Sher L. Hendrickson, James A. Lautenberger, Leslie Wei Chinn, Michael Malasky, Efe Sezgin, Lawrence A. Kingsley, James J. Goedert, Gregory D. Kirk, Edward D. Gomperts, Susan P. Buchbinder, Jennifer L. Troyer, Stephen J. O'Brien

**Affiliations:** 1 Laboratory of Genomic Diversity, National Cancer Institute, Frederick, Maryland, United States of America; 2 Laboratory of Genomic Diversity, SAIC-Frederick, Inc., Frederick, Maryland, United States of America; 3 Department of Infectious Diseases and Microbiology and Department of Epidemiology, University of Pittsburgh, Pittsburgh, Pennsylvania, United States of America; 4 Infections and Immunoepidemiology Branch, National Cancer Institute, Bethesda, Maryland, United States of America; 5 The Department of Epidemiology, Johns Hopkins Bloomberg School of Public Health, Baltimore, Maryland, United States of America; 6 Division of Hematology/Oncology, Children's Hospital of Los Angeles, Los Angeles, California, United States of America; 7 San Francisco City Clinic, Department of Public Health, San Francisco, California, United States of America; The J. Craig Venter Institute, United States of America

## Abstract

**Background:**

The human mitochondrial genome includes only 13 coding genes while nuclear-encoded genes account for 99% of proteins responsible for mitochondrial morphology, redox regulation, and energetics. Mitochondrial pathogenesis occurs in HIV patients and genetically, mitochondrial DNA haplogroups with presumed functional differences have been associated with differential AIDS progression.

**Methodology/Principal Findings:**

Here we explore whether single nucleotide polymorphisms (SNPs) within 904 of the estimated 1,500 genes that specify nuclear-encoded mitochondrial proteins (NEMPs) influence AIDS progression among HIV-1 infected patients. We examined NEMPs for association with the rate of AIDS progression using genotypes generated by an Affymetrix 6.0 genotyping array of 1,455 European American patients from five US AIDS cohorts. Successfully genotyped SNPs gave 50% or better haplotype coverage for 679 of known NEMP genes. With a Bonferroni adjustment for the number of genes and tests examined, multiple SNPs within two NEMP genes showed significant association with AIDS progression: *acyl-CoA synthetase medium-chain family member 4* (*ACSM4*) on chromosome 12 and *peroxisomal D3,D2-enoyl-CoA isomerase* (*PECI*) on chromosome 6.

**Conclusions:**

Our previous studies on mitochondrial DNA showed that European haplogroups with presumed functional differences were associated with AIDS progression and HAART mediated adverse events. The modest influences of nuclear-encoded mitochondrial genes found in the current study add support to the idea that mitochondrial function plays a role in AIDS pathogenesis.

## Introduction

The main function of mitochondria is to produce energy via oxidative phosphorylation (OXPHOS); however, they also perform several other tasks critical for proper cell functioning including regulation of apoptosis, production of radical oxygen species (ROS), and other cell-type specific metabolic processes such as cholesterol biosynthesis or hormone production. Thus, if mitochondria processes are disrupted, not only energy production, but also cell-specific products needed for normal cell functioning will be affected. Research on HIV-infected CD4-T cells indicates that the virus disrupts a number of mitochondrial pathways including cholesterol biosynthesis, OXPHOS, cellular integrity, and apoptosis [1], [2], [3], [4], [5], [6]. Proteins encoded by HIV, including Vpr, Nef, Vif, Vpu, Tat, and Rev, initially impede programmed cell death, allowing for proliferation of HIV-infected cells, then gradually shift to actively induce apoptosis of CD4-T cells, macrophages, monocytes, and microglial cells, thus causing the hallmark global immunodefiency of AIDS [7]. Systematic mitochondrial dysfunction is also evident during AIDS progression as mtDNA depletion [8], increased ROS production [9], antioxidant enzyme deficiency [10], and increased oxidative damage among patients with accelerated disease [11].

The mitochondrial genome encodes 13 proteins that are vital to mitochondrial bioenergetics. We found in earlier studies that mitochondrial DNA genotypes carrying mutations in these genes are associated with rate of AIDS progression in untreated patients [12] and the severity of lipoatrophy in patients on highly active antiretroviral therapy (HAART) [13]. Approximately 1,500 additional nuclear-encoded genes have been identified to account for the remaining protein machinery responsible for mitochondrial morphology, redox regulation and energetics [14]. Recently, over 1000 nuclear-encoded mitochondrial proteins (NEMPs) were identified in humans through a comprehensive approach utilizing both proteomics and bioinformatics [15]. It may be relevant that 4–8% of siRNAs [16], [17], [18] and 14–40% of proteins [19], [20], [21] that have been identified as host factors involved in HIV infection are also NEMPs, suggesting that NEMPs may be overrepresented among cellular factors involved with HIV. Here we surveyed single nucleotide polymorphism (SNP) variants within and around 904 known human NEMPs as potential AIDS restriction genes (ARGs). We genotyped 1455 patients of European descent from five longitudinal US AIDS cohorts, asking whether SNPs within NEMP loci are associated with AIDS progression.

## Methods

### Cohorts

The study group consisted of total of 1455 patients of European ancestry from five longitudinal cohorts including the Multicenter AIDS Cohort Study (MACS) [22], the San Francisco City Clinic Study (SFCC) [23], Hemophilia Growth and Development Study (HGDS) [24], the Multicenter Hemophilia Cohort Study (MHCS)[25], and the AIDS Linked to Intravenous Experiences (ALIVE) cohort (Table S1) [26]. Informed written consent was obtained from all patients. HIV/AIDS-related genetic studies of the cohorts has been granted by the NIH Office of Human Subjects Research under # 4010 (OHSR #4010). Ninety-seven percent of patients were male. There are two cohorts of subjects with hemophilia who contracted AIDS principally through exposure to contaminated blood products between 1978 and 1983: the MHCS is a multi-center longitudinal cohort study enrolling subjects from 17 American or European treatment centers beginning in September 1982 and HGDS is a US-based multicenter cohort of participants from 14 US treatment centers who became infected between 1982–1983. Sexual transmission in men who have sex with men is the primary mode of HIV infection for MACS and SFCC, while intravenous drug use is the principal mode of transmission in ALIVE.

### Quality Control for the Affymetrix 6.0 genotyping array

#### Subjects

All DNA samples that passed Contrast QC (CQC>0.4) were genotyped using Affymetrix Power Tools (apt-probeset-genotype 1.10.0). In addition, 4 controls run a total of 39 times, 145 duplicate samples and 8 CEPH parent-offspring trios were included. Samples were originally genotyped by batch (200–500 samples), and individuals with less than 90% call rates were excluded from further analysis. Duplicates and CEPH controls run within and between plates were verified for concordance across runs (average concordance 98.8% for all SNPs across all plates). In addition, once acceptable genotyping standards were met, Affymetrix genotypes were compared to previously produced Taqman, Illumina, and/or Perlegen genotypes at an average of 85 (range: 10–142) sites/sample. Samples with <90% concordance with their previous genotypes were discarded. Finally, Affymetrix gender calls (based on Y chromosome markers, X heterozygosity, and intensity of the sex chromosome invariant probes) were compared to clinical data files and inconsistent individuals were discarded.

Final subject QC was performed in PLINK to determine cryptic identity and cryptic relatedness and to double-check mistaken gender calls. Identical subjects (pi-hat >0.9) were investigated and either discarded (if clinical data did not match), or reduced to a single genotype file if clinical data, including birth date, matched. One individual was found who was enrolled in two cohorts. Related subjects were flagged, but not removed.

#### Genotyping

All individuals that passed initial QC were re-clustered together and SNPS were interrogated on the Affymetrix 6.0 genotyping platform. Autosomal SNPs supported by Affymetrix (871,553 total SNPs) were evaluated for Mendelian inheritance with 8 CEPH trios, SNP call rates of >95%, and Hardy Weinburg equilibrium (HWE; p>0.001). A minor allele frequency cutoff of 0.01 was also imposed. Finally, the 19,951 SNPS that failed HWE were visually assessed to approve, correct, or exclude genotyping calls based on the quality of the genotyping clusters. Of these, 2,391 were acceptable, 9,863 were manually adjusted to correct the calls, and the remaining 7,967 SNPs were excluded, resulting in a total of 700,022 SNPs approved in European Americans. None of the significant associations discussed in this study violated HWE.

### NEMP gene coverage Affymetrix 6.0 genotyping array

After subjects and SNP passing QC were re-clustered and recalled; average call rate was 99.3% with a minimum of 94.04% [27]. A total of 10,012 successfully genotyped SNPs that were within 5 kB of 904 NEMP genes (out of 966 NEMP genes found on chromosomes 1–22) as shown as Table S2.

844 of the NEMP genes were covered by the International HapMap [28]. Haplotype coverage of the NEMP genes on the Affymetrix genome scan was determined with Tagger (http://www.broadinstitute.org/mpg/tagger) evaluation using HapMap release 21 from the International HapMap Project, and build 35/UCSC hg17/May 2004 coordinates for genes and tag SNPs [29]. All SNPs that were successfully genotyped on the Affymetrix 6.0 genotyping arrays located within 5 Kb up, and 1 Kb downstream of the gene were evaluated in order to capture any potential upstream promoters or linkage surrounding the gene. SNP coverage was >50% for 679 genes, and 421 of these had coverage >80% and of these, 257 had coverage >90% (Table S3).

### Analyses

All individual follow-up data were censored at time of HAART availability. AIDS morbidity was defined with the AIDS-1987 Center for Disease Control definition [30] (HIV-1 infection plus AIDS defining illness). All SNPs were tested as additive, dominant and recessive genetic models. Tests for association were performed using both categorical case-control and Cox proportional hazards models [31]. For survivorship models, only known seroconvertors (SC) (N = 703 European Americans) were used for analysis. Seroconversion date was defined as the midpoint between seronegative and seropositive test results, which had to occur within 3 years of one another. To improve power and include valuable information from an additional 444 seroprevalent (SP) slow progressors in our study, we also performed categorical analyses between rapid and slow progressors, defined as <10 years or > = 10 years before development of clinical AIDS (AIDS-1987 definition). A Fisher's Exact test was used for dichotomous categorical analyses with the recessive and dominant genetic models and a Mantel-Haenszel test statistic was used for the additive model. Cox proportional hazard models were stratified by age at seroconversion (0–20, 20–40 and >40 years) and *p*-values were computed with the log-likelihood test statistic.

Genetic association analyses were performed with SAS version 9.1 (SAS Institute, Inc, Cary NC). Two statistical significance levels were examined. The first (GT) adjusted for the number of genes analyzed in the study multiplied by the two hypotheses and three genetic models (*p*_GT_; threshold α = 0.05/904×6 = 9.2×10^−6^). This measure is likely anti-conservative due to multiple SNPs in each gene. The second correction (ST) adjusted for the total SNPs (10,012) and the six tests outlined above (*p*_ST_ α = 0.05/10012×6 = 8.3×10^−7^); however this correction is likely overly conservative since prevalent linkage disequilibrium between adjacent SNPs means loci are not independent. The precise correction likely falls somewhere between these two measures; therefore we report both *p*-values to serve as guidelines for evaluating the association data.

For genetic variations discovered to be associated with progression to AIDS-1987, sequelae including Kaposi's sarcoma (KS) and *Pneumocystis carinii* pneumonia (PCP) were tested in dichotomous case-case categorical and survival models. Controls included AIDS patients who developed an AIDS-1987 sequelae other than the sequela of interest. Cases were defined both as those who developed a particular sequela, and as those who developed that sequela as their first AIDS-1987 defining condition. This second approach reduces the potential confounding that developing an initial AIDS defining disease may increase the risk of developing a second one [32].

### Identification and correction for population structure/stratification among NEMP SNPs

Two most significant eigen vectors based on 50K distantly spaced autosomal SNPs (excluding mitochondrial, X and Y chromosome SNPs, and SNPs out of HWE or in linkage disequilibrium) were calculated for 1455 European American samples using the Principal Components Analysis module of *Eigensoft* software [33]. Upon analysis with CEPH –HapMap samples and comparison of distribution of European Americans from this study to that of previously published reports [33], [34] we inferred that these two eigen axes correlated with genetic ancestry (Figure S1). The distribution of sero-status (SC, SP) and AIDS outcomes were compared with these recovered significant eigen axes, and no significant difference between different sero-statuses, or cases and controls in AIDS outcomes were found.

We further estimated the contribution of individual NEMP SNPs to the observed population substructure in European American samples using linear models:
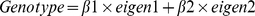



The significance of each slope corresponded to the significance of the trend observed for a given SNP's distribution along the eigen vectors. If a SNP was highly structured and did not indicate any AIDS outcome association in an unadjusted test result, then that SNP was excluded from further analyses. If a SNP was highly structured and indicated a potential AIDS outcome association, then the eigen vectors were used as covariates in the applied tests. None of the significant genetic variants discussed in this study showed significant population substructure. Further, all SNPs showing significant AIDS outcome results were reanalyzed using eigen vectors as covariates in the association tests. The eigen vector adjustments did not make any significant difference on the association test results. Unadjusted test results are presented throughout the manuscript.

## Results

### Associations between NEMPs and AIDS progression

**Figure 1:**
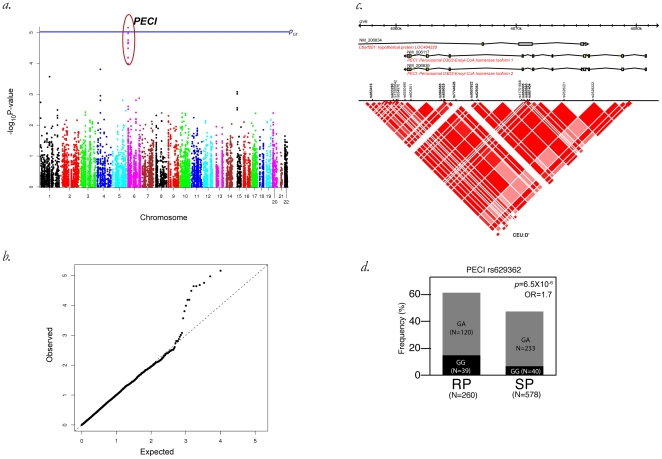
Association of SNPs within the PECI gene region of chromosome 6 with AIDS 1987. A Manhattan plot (a.) and corresponding QQ plot (b.) for 10012 SNPs in NEMP genes showing the additive model that implicates SNPs in the PECI gene region exceeding the GT significance threshold (horizontal line, 9.2×10^−6^). A gene view of PECI showing SNPs tested and corresponding linkage disequilibrium as inferred by D' between SNPs (c.) and a bar-plot of genotypes of PECI versus the AIDS-1987 diagnosis in years past seroconversion (d.) are shown.

**Figure 2:**
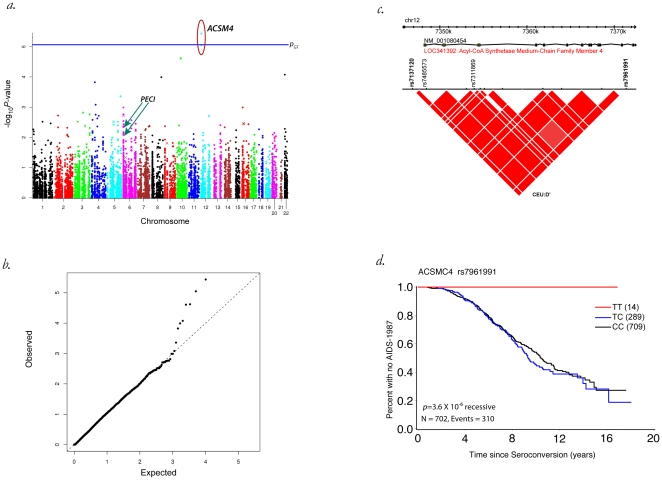
Association of SNPs within the ACSM4 gene region of chromosome 12 with AIDS 1987. Shown as a a) Manhattan plot for time to AIDS-1987 (genetic model-recessive, GT significance threshold is shown as a horizontal line at 9.2×10^−6^), b) QQ plot, c) gene view of ACSM4 showing SNPs tested and corresponding linkage disequilibrium as inferred by D' between SNPs, and d) Kaplan-Meier plot of time to AIDS-1987 showing the three ACSM4 genotypes and the p-value for the recessive model.

We tested 10,012 SNPs within 904 NEMP genes for association with progression to AIDS-1987. Successfully genotyped SNPs gave 50% or better haplotype coverage for 679 of known NEMP genes. SNPs within two NEMP genes displayed significant associations; *Peroxisomal D3,D2-Enoyl-CoA Isomerase* (*PECI*) on chromosome 6 and *Acyl-CoA Synthetase Medium-Chain Family Member 4* (*ACSM4*) on chromosome 12 (Figures 1 and 2). A Manhattan plot and a QQ-plot for a dichotomous categorical analysis of AIDS-1987 in the additive model implies that several SNPs within the gene *PECI* were significantly associated with rate of AIDS progression (Figure 1; Table 1). Linkage disequilibrium between these SNPs is between D' = 0.95 for rs584585 and 1 for all others (Figure 1c). The lowest *p*-value was in observed for SNP rs629362 (*p*_unadjusted_ = 6.5×10^−6^) (Table 1). SNP rs629362 remained significant when corrected for the number of genes and tests (*p*_GT_ = 0.04), however it was no longer significant when alpha was adjusted for the number of SNPs (*p*_ST_>0.05, Table 1).

**Table 1:** SNPs within the NEMP genes that included significant association with time to AIDS-1987 CDC definition [30].

gene	entrez	chr	score	rs	pos	loc	MAF	No. SNPs	test	model	OR/RH	*p*_unadjusted_	*p_GT_*	*p_ST_*
*PECI*	10455	6	90.5	rs629362	4066830	intron	0.31	15	DCA	A	1.7	6.52×10^−6^	**0.04**	0.39
				rs853416	4058046	−2880 5′	0.32	15	DCA	A	1.6	3.25×10^−5^	0.18	1
				rs633290	4059787	−1139 5′	0.31	15	DCA	A	1.6	1.66×10^−5^	0.09	1
				rs584585	4063788	intron	0.31	15	DCA	A	1.6	1.00×10^−5^	**0.05**	0.60
				rs659025	4064050	intron	0.31	15	DCA	A	1.6	6.12×10^−5^	0.33	1
				rs7744628	4064995	intron	0.31	15	DCA	A	1.6	2.15×10^−5^	0.12	1
				rs9503922	4066332	intron	0.26	15	DCA	A	1.6	9.52×10^−5^	0.52	1
				rs659305	4070654	intron	0.31	15	DCA	A	1.6	1.96×10^−5^	0.11	1
				rs660560	4070940	intron	0.31	15	DCA	A	1.6	2.15×10^−5^	0.12	1
				rs661404	4071096	intron	0.40	15	DCA	A	1.5	6.40×10^−5^	0.35	0.35
														
*ACSM4*	341392	12	75	rs7961991	7371395	intron	0.16	4	PH	R	Und	3.59×10^−6^	**0.02**	0.22
				rs7137120	7346864	−1331 5′	0.16	4	PH	R	Und	8.84×10^−6^	**0.05**	0.53

Results are from proportional hazards (PH) models or dichotomous categorical analyses (DCA). Chr = chromosome. Score is the haplotype coverage generated by Tagger with the number of SNPs shown for that gene (SNPs) [29]. Pos =  the position from HapMap Project, and build 36. loc =  location relative to the gene. MAF =  Minor allele frequency. R = recessive genetic Model, A = additive genetic model. RH = relative hazard. If the analysis was a DCA, or categorical test, this equates to the Odds Ratio (OR). Und = undefined, which means that there were no cases that succumbed to disease and therefore the value is not statistical calculable. *p*_GT_ is the *p-*value corrected for 904 genes and six tests. *p*_ST_ is the *p*-value corrected for the number of SNPs and the six tests (see methods).

A strong association was also observed for rs7961991 in *ACSM4* in survivorship analyses (p = 3.6×10-6, Table 1, Figure 1). This SNP remained significant when the *p*-value was corrected for the number of genes and tests *(p*_G*T*_ = 0.02), but was no longer significant when the number of SNPs and tests (*p*_ST_) were corrected for (*p*_ST_ = 0.2). A Manhattan plot for time to AIDS-1987 (recessive genetic model) and the corresponding QQ plot are shown in figure 2a and 2b respectively. The SNPs in the *ACSM4* gene were in complete LD (D' = 1) as shown in Figure 2c. We see that only two of the four SNPs in this gene were significant, suggesting the significant SNPs were likely tracking a yet to be discovered causal functional SNP in the region of strong LD. All SNPs with significant associations were in introns, or upstream of the gene as indicated in Table 1.

### Associations between PECI and ASCM4 and mitochondrial DNA haplogroups

In a previous study, we found that certain mitochondrial DNA (mtDNA) haplogroups were associated with AIDS progression [12]. Because of the phylo-geographic structure of mitochondrial DNA haplogroups and the potential for epistatis between NEMPs and the mitochondrial genome, the significant genotypes in *PECI* and *ASCM4* were checked for epistatic interaction with mitochondrial DNA haplogroups with a Mantel-Haenszel Chi-square test. No associations were observed between *PECI* SNP rs629362 and haplogroups H, J, T, U, W, X, or N1I (Table 2). Haplogroup X was associated with the *ASCM4* SNP rs7961991 (*p* = 0.009); however, out of 22 individuals in haplogroup X, only one person had the recessive genotype associated with AIDS progression in this study, and the adjusted Bonferroni *p* was >0.05. None of the SNPs were found to have significant geographic structure.

**Table 2:** Mitochondrial DNA haplogroup associations with highly significant SNPs in the PECI and ASCM4 genes.

		P-value for Association with Mitochondrial DNA Haplogroup (Mantel-Haenszel Chi-Square Test - Additive Model)							
Gene	SNP	H	J	I	N1I	T	U	W	X
PECI	rs629362	0.8849	0.7312	0.7500	0.8794	0.9503	0.5194	0.8786	0.8405
ASCM4	rs7961991	0.5018	0.4424	0.1310	0.4882	0.4739	0.3474	0.1200	0.0094

Only ASCM4 was significant with haplogroup X, however X included only 22 individuals, therefore it is unlikely to explain the associations observed.

### *PECI*, *ASCM4* and AIDS sequelae

**Figure 3:**
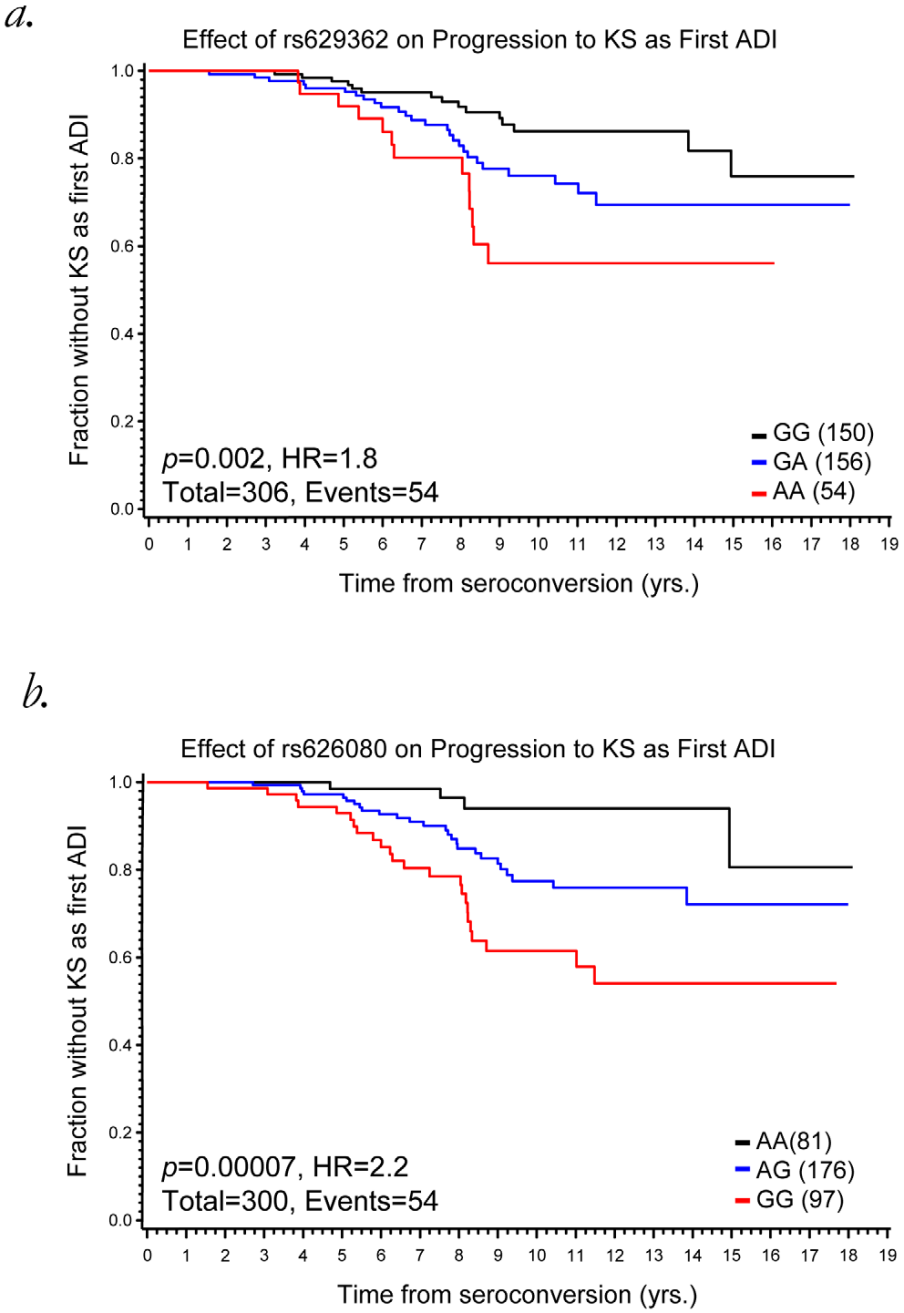
The effect of rs629362 (a) and rs626080 (b) in PECI on progression to AIDS-defining illness (ADI) Kaposi Sarcoma (KS).

SNPs within PECI, including rs629362 (p = 0.002, HR = 1.8) and rs626080 (p = 0.00007, HR = 2.2) had a strong association with KS as shown in Figure 3. No significant results were observed for KS or PCP with SNPS in *ASCM4.* Unfortunately, we lacked sufficient data to look into other less frequent sequelae.

### Mitochondrial genes and previously published studies

We further examined NEMPs that were previously reported as cellular gene products required for HIV-infection in screens using siRNAs [16], [17], [18], mRNA expression [21], or proteomics [19], [20] for SNPs associated with AIDS-1987 (Table S4). In our analysis of progression to AIDS, no SNPs within the 151 NEMP genes that were identified by the HIV infection studies [16], [17], [18] showed *p*-values above the *p*_GT_ correction threshold; however, fifty-nine genetic associations from twenty genes produce unadjusted *p*≤0.01 with the lowest *p*-value (0.0009) found in the gene for quinoid dihydropteridine reductase (QDPR) (rs2535228) for time to AIDS-1987 (HR = 0.7); six other SNPs in this region showed *p*-values from 0.004–0.01 (Table S5). SNPs within three of the gene fifteen genes replicated in two or more studies were associated with accelerated progression to AIDS-1987 in the current study: *NADH Dehydrogenase (Ubiquinone) 1 Beta Subcomplex, 7* (*NDUFB7*), *Isocitrate Dehydrogenase 1* (*IDH1*) , and *Isocitrate Dehydrogenase 3 (NAD+) Alpha* (*IDH3A*)] (NDUFB7 rs6511939 HR = 1.6, *p* = 0.008; IDH1 rs7580715 HR = 2.1, *p* = 0.009, IDH3A rs11855354, rs8032618 and rs12903696 HR = 1.6, p = 0.007–0.009).

## Discussion

We examined 904 genes for nuclear-encoded mitochondrial proteins for association with the rate of AIDS progression using genotypes generated by an Affymetrix 6.0 genotyping array of 1455 European American patients from five US AIDS cohorts. With a Bonferroni adjustment for the number of genes and tests examined, multiple SNPs within two NEMP genes showed significant association with AIDS progression: *acyl-CoA synthetase medium-chain family member 4* (*ACSM4*) on chromosome 12 and *peroxisomal D3,D2-enoyl-CoA isomerase* (*PECI*) on chromosome 6.

Antibodies to PECI have been found in patients with autoimmune diabetes [35], breast cancer[36], renal cancer [37], and hepatocarcinoma [38] and PECI serves as an autoantigen eliciting immune attack against hematopoietic progenitor cells by both T and B cells in acquired aplastic anemia patients [39]. PECI is an auxiliary enzyme that catalyzes an isomerization step required for the beta-oxidation of unsaturated fatty acids [39], [40]. Cellular energy metabolism is largely sustained by mitochondrial β-oxidation of saturated and unsaturated fatty acids. A recent study in the THESA cohort suggested that intake of poly-unsaturated fats was adversely related to liver function in asymptomatic HIV-infected subjects compared with HIV-uninfected subjects [41]. One hypothesis put forth by the THESA study is that polyunsaturated fats, which are susceptible to attack by free radicals and oxidation into lipid peroxides, promote liver damage because of increased oxidative stress in HIV-infected subjects. This hypothesis will need to be further explored to resolve if and how the associations observed between SNPs in *PECI* and accelerated AIDS progression here may be related.

ACSM4 is a member of a group of synthases that catalyze the fundamental initial reaction in fatty acid metabolism. This activation of fatty acids allows their participation in both anabolic and catabolic pathways [42]. How these functions may relate to AIDS progression remains to be discovered. As ACSM4 associations are weaker and lack a obvious mechanism, we consider these associations preliminary.

AIDS'87 is defined by diagnosis of an AIDS sequela, or “clinical” AIDS, therefore most patients are typically past the earlier CD4 cell count <200 stage. To look for underlying associations between *PECI* and *ASCM4* and AIDS-1987 defining conditions, KS, PCP and opportunistic infections were analyzed for association with significant SNPs in these two genes. These data suggest the connection between PECI and AIDS progression may be through an association to KS.

It should be noted, that although these tests were follow-up to the genes found significant in progression analyses, if a strict Bonferoni criteria considering all tests was used, these results would no longer be significant. We have tried to account for this matter by using two methods, the first (GT) adjusted for the number of genes analyzed in the study multiplied by the two hypotheses and three genetic models, and a second correction (ST) adjusted for the total SNPs (10,012) and the six tests. The precise correction likely falls somewhere between these two measures; as the first is not conservative enough, while the second is likely too strict. P-values as presented are moderate and conclusions must be tempered by the multiple comparisons problem. Replication of these genes in additional cohorts is necessary.We also examined NEMPs that were previously reported as cellular gene products required for HIV-infection in screens using siRNAs [16], [17], [18], mRNA expression [21], or proteomics [19], [20] for SNPs associated with AIDS-1987. Out of 1353 cellular factors identified in these studies, 151 are also identified NEMPs. NEMP genes are significantly overrepresented in Chan *et al*. 2007, Ryo *et al.* 2000 and in Ringrose *et al.* 2008, and a trend is observed in Espeseth *et al.* 2008 (Table S3), suggesting a strong link between mitochondrial function and HIV infection. None of the SNPs within the 151 NEMP genes that were identified by the HIV infection studies showed *p*-values above the *p*_GT_ correction threshold; however, fifty-nine genetic associations from twenty genes produce unadjusted *p*≤0.01, with the strongest significance found for quinoid dihydropteridine reductase (QDPR) (rs2535228). SNPs within three of fifteen genes replicated between the original studies were associated with accelerated progression to AIDS-1987 in the current study: *NADH Dehydrogenase (Ubiquinone) 1 Beta Subcomplex, 7* (*NDUFB7*), *Isocitrate Dehydrogenase 1* (*IDH1*) , and *Isocitrate Dehydrogenase 3 (NAD+) Alpha* (*IDH3A*)]. These associations may be real or may be statistical artifacts of multiple tests, therefore they need to be validated in other studies. However, s, the independent replication of these three HIV factors, plus the observation of modest SNP associations for these genes with AIDS progression adds support to a plausible regulatory role of each NEMP-HIV factor occurs in HIV-related pathogenesis.

### Conclusion

Our previous studies on mitochondrial DNA that showed European haplogroups with presumed functional differences were associated with AIDS progression and HAART mediated adverse events [12], [13], and the modest influences of nuclear-encoded mitochondrial genes found in the current study add support to the idea that mitochondrial function plays a role in AIDS pathogenesis.

## Supporting Information

Figure S1 First and second principal components from Eigensoft analysis of SNPs spaced every 50K across the entire genome.
(1.15 MB EPS)

Table S1 Clinical cohorts and the number of seroconverters, seroprevalents and HIV-exposed seronegatives.
(0.03 MB DOC)

Table S2 NEMP genes and SNPS included in current study.
(2.09 MB XLS)

Table S3 Haplotype tagging coverage scores of 1000 human NEMP genes on the Affymetrix 6.0 genome scan.
(0.03 MB DOC)

Table S4 Nuclear-encoded proteins in previous mRNA and proteomics studies of HIV-dependant factors.
(0.03 MB DOC)

Table S5 SNPs associated with AIDS-1987 progression with p<0.01 in our study that have been identified as HIV-dependant factors in prior mRNA and proteomic studies.
(0.04 MB XLS)
