# Supplementary material for: Genetic Variants in Nuclear-Encoded Mitochondrial Genes Influence AIDS Progression
Source: PLoS One. 2010 Sep 21;5(9):e12862. doi: 10.1371/journal.pone.0012862 (PMC2943476; doi:10.1371/journal.pone.0012862)
Supplement: Table S1 — Clinical cohorts and the number of seroconverters, seroprevalents and HIV-exposed seronegatives. (0.03 MB DOC) [file pone.0012862.s002.doc]

Table S1. Clinical cohorts and the number of seroconverters, seroprevalents and HIV-exposed seronegatives.

| Cohort | Seronegative | Seroconverter | Seroprevalent | Total |
| --- | --- | --- | --- | --- |
| ALIVE | 1 | 13 | 3 | 17 |
| DCG | 1 | 38 | 2 | 41 |
| HGDS | 11 | 0 | 30 | 41 |
| MACS | 143 | 407 | 293 | 843 |
| MHCS | 99 | 169 | 89 | 357 |
| SFCC | 4 | 76 | 76 | 156 |
|  | 259 | 703 | 493 | 1455 |
