# Supplementary material for: Genetic Variants in Nuclear-Encoded Mitochondrial Genes Influence AIDS Progression
Source: PLoS One. 2010 Sep 21;5(9):e12862. doi: 10.1371/journal.pone.0012862 (PMC2943476; doi:10.1371/journal.pone.0012862)
Supplement: Table S3 — Haplotype tagging coverage scores of 1000 human NEMP genes on the Affymetrix 6.0 genome scan. (0.03 MB DOC) [file pone.0012862.s004.doc]

Table S3. Haplotype tagging coverage scores of 1000 human NEMP genes on the Affymetrix 6.0 genome scan estimated in Tagger (11). 157 genes were either not covered on the GWAS, or they did not have representation on HapMap Build 35.

|  | **Haplotype coverage (%)** | | | | | | | |  |
| --- | --- | --- | --- | --- | --- | --- | --- | --- | --- |
|  | **100** | **≥90** | **≥80** | **≥70** | **≥60** | **≥50** | **<50** | **?** | |
| Genes | 96 | 257 | 421 | 530 | 606 | 679 | 164 | 157 | |
