# Supplementary material for: Genetic Variants in Nuclear-Encoded Mitochondrial Genes Influence AIDS Progression
Source: PLoS One. 2010 Sep 21;5(9):e12862. doi: 10.1371/journal.pone.0012862 (PMC2943476; doi:10.1371/journal.pone.0012862)
Supplement: Table S4 — Nuclear-encoded proteins in previous mRNA and proteomics studies of HIV-dependant factors. (0.03 MB DOC) [file pone.0012862.s005.doc]

Table S4. Nuclear-encoded proteins in previous mRNA and proteomics studies of HIV-dependant factors. The Chi-square test is based on a estimate of finding NEMPs in the genome at a frequency of 0.05% (1000 known out of 20,000 total human genes). NEMPs were identified with the MitoCarta list (1000 NEMP genes).

|  | **siRNA** | | | **Proteomics** | | |
| --- | --- | --- | --- | --- | --- | --- |
|  | Espeseth *et al.* 2008 | Brass *et al.* 2008 | Chandra *et al.* 2007 | Chan *et al.* 2007 | Ryo *et al.* 2000 | Ringrose *et al.* 2008 |
| Total N reported | 231 | 280 | 293 | 517 | 48 | 88 |
| N NEMPs | 18 | 11 | 18 | 74 | 9 | 35 |
| Percent NEMPs | 8% | 4% | 6% | 14% | 19% | 40% |
| N NEMPs expected | 12 | 14 | 15 | 26 | 2 | 4 |
| ℵ^2^ | 0.08 | 0.4 | 0.4 | <0.0001 | <0.0001 | <0.0001 |
